# A Low-Cost Inertial Measurement Unit Motion Capture System for Operation Posture Collection and Recognition

**DOI:** 10.3390/s24020686

**Published:** 2024-01-21

**Authors:** Mingyue Yin, Jianguang Li, Tiancong Wang

**Affiliations:** 1 School of Mechatronics Engineering, Harbin Institute of Technology, Harbin 150001, China; 21b908117@stu.hit.edu.cn; 2 School of Astronautics, Harbin Institute of Technology, Harbin 150001, China; 17862700589@163.com

**Keywords:** motion capture, inertial measurement unit, human posture recognition, BiLSTM model

## Abstract

In factories, human posture recognition facilitates human–machine collaboration, human risk management, and workflow improvement. Compared to optical sensors, inertial sensors have the advantages of portability and resistance to obstruction, making them suitable for factories. However, existing product-level inertial sensing solutions are generally expensive. This paper proposes a low-cost human motion capture system based on BMI 160, a type of six-axis inertial measurement unit (IMU). Based on WIFI communication, the collected data are processed to obtain the displacement of human joints’ rotation angles around XYZ directions and the displacement in XYZ directions, then the human skeleton hierarchical relationship was combined to calculate the real-time human posture. Furthermore, the digital human model was been established on Unity3D to synchronously visualize and present human movements. We simulated assembly operations in a virtual reality environment for human posture data collection and posture recognition experiments. Six inertial sensors were placed on the chest, waist, knee joints, and ankle joints of both legs. There were 16,067 labeled samples obtained for posture recognition model training, and the accumulated displacement and the rotation angle of six joints in the three directions were used as input features. The bi-directional long short-term memory (BiLSTM) model was used to identify seven common operation postures: standing, slightly bending, deep bending, half-squatting, squatting, sitting, and supine, with an average accuracy of 98.24%. According to the experiment result, the proposed method could be used to develop a low-cost and effective solution to human posture recognition for factory operation.

## 1. Introduction

In the era of Industry 4.0, motion capture systems will find broader applications in engineering for digital human modeling [1]. In the factory, the recognition of human body movement contributes to human–machine collaboration [2] and human factor analysis [3]. In contrast to optical cameras, inertial sensors are more flexible and resistant to obstruction, making them suitable for scenarios such as automotive assembly [4,5]. Researchers have established methods for capturing full-body motion by sparse inertial sensors. Susperregi et al. [6] proposed the fusion of multiple low-cost sensors and cameras to capture human behavior, addressing data bias through data fusion. Caputo et al. [4] utilized a motion capture system to estimate the basic segment positions of the human body. He et al. [7] introduced a wavelet tensor fuzzy clustering scheme for analyzing multisensor signals to capture human behavior, achieving higher recognition accuracy compared to the fuzzy mean clustering method. Liu et al. [8] developed a segmentation procedure based on a moving average window algorithm, and introduced a double-threshold technique for automatic recognition and segmentation of calibration postures. Yi et al. [9] tracked human motion using only six inertial sensors, combining a neural kinematic estimator and a physical perception motion optimizer. Previous work has provided good guidance for achieving low-cost inertial sensor dynamic capture, giving IMUs potential application prospects in the engineering field.

Due to the inevitable presence of a large number of metal objects in the factory environment, the negative impact on magnetometers needs to be considered. Therefore, we have chosen the cost-effective six-axis sensor chip BMI160, along with the ESP8266-NodeMCU chip, IP5306 BMS charging board, and li-ion battery, to collectively form our tracker; the total cost is USD 3.60. A comparison of prices and performance of other IMU solutions is presented in Table 1, covering aspects such as price, sampling rate, accelerometer rate noise spectral density, gyroscope rate noise spectral density, interface mode, and battery life. The IMU solutions commonly used in human motion recognition research, such as Xsens MTw Awinda [10], MetaMotionR [10], Next-Generation IMU [11,12], MetaMotionC [13], Shimmer3 [14], and InvenSense MPU-9250 [15], were chosen. Through comparison, our solution demonstrates advantages in terms of pricing. Moreover, for the collection of human body movements, the sampling rate and accuracy within our solution fall within acceptable ranges, and the interface and the battery life employed in our solution are also sufficient for human motion capture.

Machine learning is commonly used in human motion recognition research, for example, the support vector machines classification model [16], Markov model [17], and random forest (RF) [18]. In the past few years, deep learning algorithms have found extensive applications in the realm of human motion recognition [19], demonstrating superior recognition performance compared to traditional algorithms [20,21]. Akkaladevi et al. [22] proposed a multilabel human action recognition framework using a spatiotemporal graph convolutional network (ST-GCN) to capture spatial and temporal relationships between joint sequences. Tang et al. [23] introduced a novel dual-branch interactive network (DIN) that incorporates the strengths of both CNNs and transformers for managing multichannel time series. Wang et al. [24] explored adaptive networks that can dynamically adjust their structure based on available computing resources, allowing for a trade-off between accuracy and speed. Dey et al. [25] utilized a three-layer stacked temporal convolutional network to predict foot angular positions. Oh et al. [26] employed a pattern recognition method based on an artificial neural network algorithm to detect different gait states. Seenath et al. [27] proposed the conformer-based human activity recognition model, which leverages attention mechanisms to better capture the temporal dynamics of human motion and improve recognition accuracy. Considering that IMU motion capture data contain both temporal and spatial information, Chen et al. [28] used a deep convolutional neural network with a bidirectional long short-term memory network (DCNN-BiLSTM) to recognize and estimate four swimming styles. Based on deep learning algorithms, the accuracy of human motion recognition can reach around 90%. Based on existing research, we will carry out IMU-based human operation posture recognition.

Existing product-level inertial motion capture devices generally require high purchasing costs. This paper aims to explore a low-cost operation motion capture system and an operation posture recognition solution based on IMU. The cost-effective core components are used to build the human motion capture system, and experimental tests are conducted in virtual factory environments. A deep neural network model is used to recognize multiple basic operation postures offline based on experimental datasets. This paper is organized as follows: Section 1 offers an overview of the research status and significance of IMU-based human motion capture and operation posture recognition. Section 2 introduces the low-cost assembly operation motion capture scheme based on IMU from the aspects of hardware configuration, motion signal processing, and human motion reproduction. Section 3 describes the operation motion capture experiment and the operation posture recognition method based on the BiLSTM model. Section 4 discusses the proposed research methods and suggests future research directions; Section 5 summarizes the proposed research work.

## 2. Design of a Low-Cost Motion Capture System Based on IMU

### 2.1. Overall Solution

**Figure 1:**
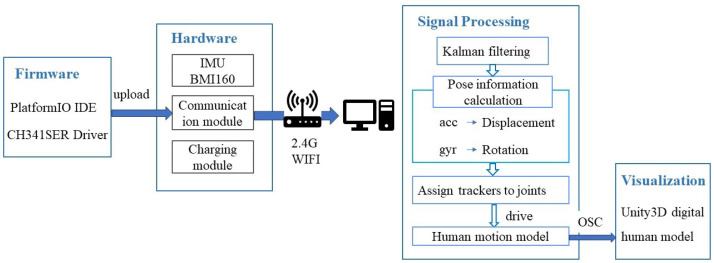
The structure of the low-cost motion capture system based on IMU.

This paper proposes a low-cost human motion capture system based on IMU. As shown in Figure 1, the system consists of four main components: a firmware module, a hardware module, a signal processing module, and a synchronized visualization module. In the hardware part, the core modules include the inertial measurement, communication, and charging modules. The inertial measurement module utilizes BMI160. The communication module uses the ESP8266 chip for wireless communication via WiFi. The charging module consists of a charging integration board, a battery, and a switch. The BMI160 is driven by the CH341SER. The firmware code is compiled and run in PlatformIO IDE (VSCode). The tracker signals are transmitted to the host computer via WiFi, where the collected data are processed to obtain the pose information of the sensors. The trackers are assigned to the corresponding joint positions of the digital human body based on their actual wearing positions. Combined with the hierarchical relationship of the human skeleton, the real-time calculation of human posture is performed. Finally, using the Open Sound Control (OSC) network transmission protocol, the system synchronously visualizes human motion through a 3D digital human model in Unity3D.

### 2.2. Hardware

**Figure 2:**
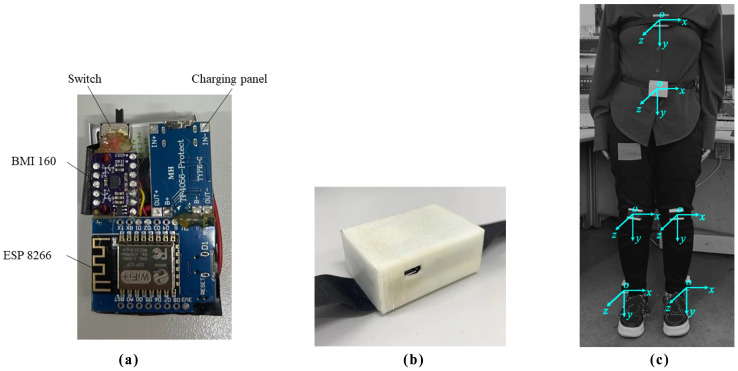
The circuit diagram and physical diagram of the tracker. (**a**) The status of completed welding. (**b**) The tracker with casing and straps attached. (**c**) The tracker placement.

The main functional components of the action tracker are the BMI160 IMU module, the ESP8266-NodeMCU module, and the IP5306 BMS charging module. Considering usability and price, the BMI160 was chosen to implement the inertial measurement function. The BMI160 chip module includes a three-axis accelerometer and a three-axis gyroscope. The chip features three 16-bit analog-to-digital converters (ADCs) for digitizing the accelerometer outputs and three 16-bit ADCs for digitizing the gyroscope outputs with standard IIC (up to 1 MHz)/SPI communication protocol. The chip can monitor an acceleration range of ±4 g and an angular velocity range of ±250°/s. The sampling rate is 100 Hz. In coordination, the ESP8266 Node MCU module, which is a version containing the ESP-12F WIFI unit with a peak power consumption of approximately 1.5 W, was selected for communication, supporting WIFI connections in the 2.4 G frequency band. Additionally, the charging module was designed using the TP4056 Type-C charging chip, an input voltage of 5 V, and a maximum charging current of 1000 mA. The 3.7 V, 1500 mAh lithium battery was chosen. Finally, two-position toggle switches were selected to control the tracker’s on and off functions. The circuit diagram and physical diagram of the tracker are shown in Figure 2a. The wires were soldered in a tightly arranged manner to minimize the size of the tracker. The tracker’s housing was 3D printed, with a total length of 54 mm, a total width of 39 mm, and a total height of 29 mm. The strap width is 25 mm, as shown in Figure 2b. In this paper, six motion trackers are used, strapped respectively to the chest, and waist, above the left knee joint, above the left ankle joint, above the right knee joint, and above the right ankle joint of the human. From top to bottom, these trackers represent the movements of the chest, waist, knee end of the femur bone, and the ankle end of the tibia bone. The wearing positions and directions of IMU are shown in Figure 2c. When wearing, the direction of the BMI160 inside each tracker is consistent, with the Y-axis pointing towards the ground and the Z-axis pointing towards the front of the body. The length values of each segment of the experimenter’s body have been pre-inputted into the terminal, and the movement status of the trunk and lower limbs can be obtained by providing joint displacement and angle. Before the motion capture experiment, the experimenter needs to make two designated postures: upright posture and skiing posture, to calibrate the initial direction of each tracker.

### 2.3. Signal Processing

The processing of motion signals involves two main parts: filtering and drift compensation of IMU signals. Kalman filtering algorithm is used for filtering. Human motion is irregular but within a certain activity space. The Kalman filtering algorithm is a classic method for processing IMU signals, which consists of predicting the position of the next time step, and correcting the position of the current state. The specific implementation principle is as follows.

(1) $${\hat{X}}_{\bar{k}}=A_{\bar{k}}{\hat{X}}_{k-1}+B_{k}u_{k}$$

(2) $$P_{\bar{k}}=A_{\bar{k}}P_{k-1}A_{k}^{T}+Q$$

Above are prediction equations. In Equation (1), ${\hat{X}}_{\bar{k}}$ represents the prior state estimation at time *k*, and ${\hat{X}}_{k-1}$ represents the posterior state at time *k*−1, respectively. $A_{\bar{k}}$ is a transformation matrix that represents the proportion of the previous state’s correction to the current state result. $B_{k}$ represents the control variable matrix, and $u_{k}$ is the state control vector. In Equation (2), $P_{\bar{k}}$ represents the prior estimate covariance at time *k*, and $P_{k-1}$ represents the posterior estimate covariance at time k−1 , *Q* is the covariance of the system process noise.

(3) $$K_{k}=\frac{P_{\bar{k}}H_{k}^{T}}{H_{k}P_{\bar{k}}H_{k}^{T}+R}$$

(4) $${\hat{X}}_{k}={\hat{X}}_{\bar{k}}+K_{k}\left(Z_{k}-H_{k}{\hat{X}}_{\bar{k}}\right)$$

(5) $$P_{k}=\left(I-H_{k}K_{k}\right)P_{\bar{k}}$$

Equation (3) calculates the Kalman gain ( $K_{k}$ ), in which $H_{k}$ represents the prediction matrix and *R* is the covariance matrix of the measurement noise. Equation (4) uses two predicted values and a ratio to calculate the output ${\hat{X}}_{k}$ , the posterior state estimation at time *k*. $Z_{k}$ is a measurement vector. Equation (5) prepares the posteriori estimation covariance at time *k*( $P_{k}$ ) for the prediction of the next time step.

The drift compensation part mainly involves applying inverse rotation to compensate drift of the IMU. In this study, signal processing and fusion are based on the Slime VR open-source software, a recently matured open-source motion capture solution based on IMUs. Based on our experimental environment and equipment, after multiple tuning and testing sessions primarily focusing on the accuracy and stability of reproducing human motion, we finally set the filtering strength to 50% and drift compensation strength to 20%. The original signals collected by IMUs consist of XYZ tri-axis acceleration signals and XYZ tri-axis gyroscope signals. The displacement information can be obtained by integrating the acceleration signal, while the rotation angle information can be obtained by integrating the gyroscope signal.

The calculation method for obtaining the current pose from two frames of IMU data is as follows. For the acceleration data, calculate the average acceleration between the current time *t* and the next time t+1 . This average acceleration over the time interval is used to approximate the velocity and displacement at t+1 , given the initial velocity and displacement at *t*. Since the IMU acceleration data is represented in the body coordinate system, it needs to be transformed to the world coordinate system using the corresponding pose. Before the transformation, the bias needs to be subtracted, and after the transformation, the gravitational acceleration needs to be subtracted. For the gyroscope data, the average angular velocity over the time interval is calculated between *t* and the next time t+1 . With this average angular velocity and the current pose, the pose at t+1 can be approximated. Equations (6)–(12) show the entire integration process.

(6) $$a_{t,w}=Q_{t}\left(a_{t,b}-B_{a}\right)-g$$

where $a_{t,w}$ is the acceleration of IMU at time *t* in the world coordinate system, $Q_{t}$ is the quaternion of IMU at time *t*, $a_{t,b}$ is the acceleration at time *t* in the body coordinate system, $B_{a}$ is the deviation of the body coordinate system, and *g* is the gravitational acceleration.

(7) $${\bar{\omega }}_{t}=\frac{1}{2}\left(\omega_{t}+\omega_{t+1}\right)-B_{g}$$

(8) $$Q_{t+1}=Q_{t}\left(\omega_{t}\Delta t\right)$$

In Equations (7) and (8), ${\bar{\omega }}_{t}$ is the average angular velocity, $\omega_{t}$ is the angular velocity at time *t*, $\omega_{t+1}$ is the angular velocity at time t+1 , and $B_{g}$ is the gyroscope bias, $Q_{t+1}$ is IMU Quaternion at time t+1 .

(9) $$a_{t+1,w}=Q_{t+1}\left(a_{t+1,b}-B_{a}\right)-g$$

(10) $${\bar{a}}_{t,w}=\frac{1}{2}\left(a_{t,w}+a_{t+1,w}\right)$$

In Equations (9) and (10), $a_{t+1,w}$ is the acceleration in the world coordinate system at time t+1 , $a_{t+1,b}$ is the acceleration in the body coordinate system at time t+1 , and ${\bar{a}}_{t,w}$ is the average acceleration.

(11) $$V_{t+1}=V_{t}+{\bar{a}}_{t,w}\Delta t$$

(12) $$D_{t+1}=D_{t}+V_{t}\Delta t+\frac{1}{2}{\bar{a}}_{t,w}\Delta t^{2}$$

In Equations (11) and (12), $V_{t}$ is the velocity at time *t*, $V_{t+1}$ is the velocity at time *t*, $D_{t}$ is the displacement of the IMU at time *t*, and $D_{t+1}$ is the displacement of the IMU at time t+1 .

### 2.4. Online Synchronized Display of Human Body Motion

**Figure 3:**
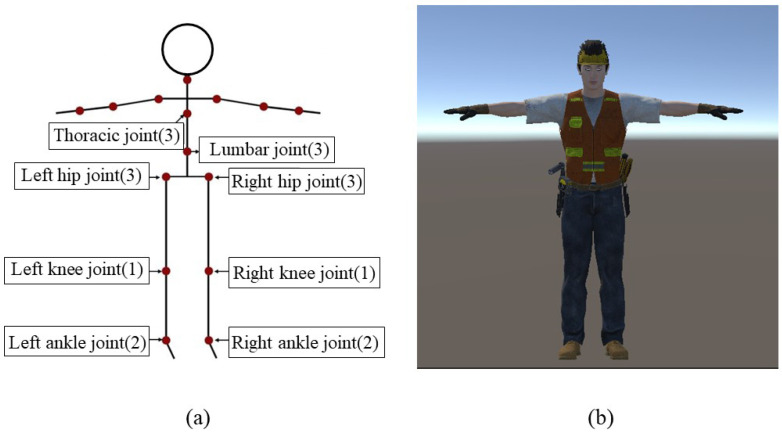
The digital human model. (**a**) Skeletal model. (**b**) Digital human model with skinning.

The online synchronized display of human body movements is achieved based on the tracker’s pose information and the hierarchical relationship of the human body skeleton. This study uses a simplified digital human model to focus on the operational movements of the human torso and lower limbs. A 3D digital human model was built on the Unity platform. The joint composition of the digital human includes thoracic joints, lumbar joints, left and right hip joints, left and right knee joints, and left and right ankle joints. In constructing the digital human model, the thoracic, lumbar, and hip joints comprise three independent subjoints capable of generating rotation, pitch, and yaw movements. The ankle joint is generally considered a ball joint with two independent axes of rotation, while the knee joint has only one axis of rotation. The head and upper limb segments are set to default states. Figure 3 shows the skeletal and digitized human models with skinning. The lengths of the body segments are set according to the experimenter’s height (1580 mm) and standard body proportions.

**Figure 4:**
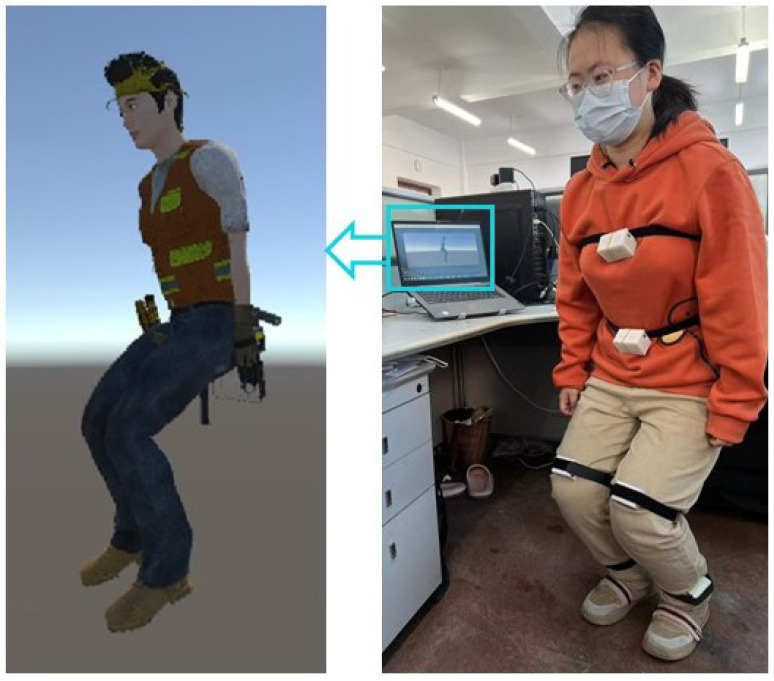
Real-time human body and digital human model.

Unity and the IMU host can communicate through the OSC protocol to achieve an online synchronized display of human body movements. Figure 4 shows the real-time human body movement at a certain moment and the corresponding movements of the digital human model at the same moment.

## 3. Human Posture Recognition with Assembly Operations

### 3.1. Basic Operation Postures

By observing the assembly and maintenance operation processes of large-scale equipment, several common assembly basic postures that facilitate exerting force could be summarized: standing posture, slightly bending posture, deep bending posture, half squatting posture, squatting posture, sitting posture, and supine posture. Operators could perform upper limb actions based on these basic postures, such as pushing (pulling), tightening (loosening), gripping, tapping, etc. The labels, names, and reference images of the basic working postures are shown in Table 2. The definition of postures mainly considered the range of bending angles of the torso, the range of bending angles of the hip joint, and the range of bending angles of the knee joint. Labels have been defined for these basic postures.

### 3.2. Operation Posture Collection Experiment

**Figure 5:**
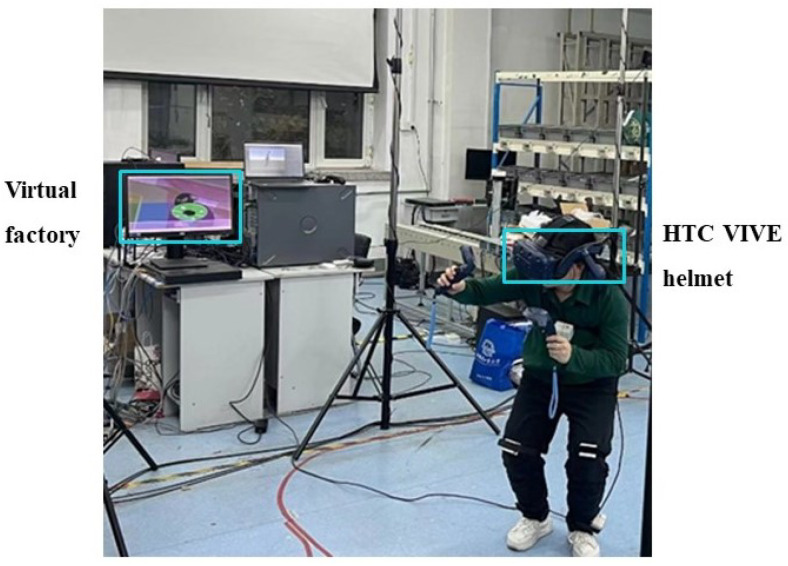
Operation posture collection experiment scene.

As shown in Figure 5, an immersive assembly scene was set up to facilitate participants making corresponding assembly movements based on prompts using Tecnomatix software and HTC VIVE devices. The participant wearing the tracker completed the operation tasks under instructions. The router was not connected to other devices to obtain sufficient bandwidth during the experiment, and the entire experiment process was recorded. At the same time, we tried to avoid other 2.4 G signals to prevent excessive data transmission delay caused by frequency congestion in the experimental environment. The experiment was conducted within a radius of 5 m from the router to ensure low data transmission delay. The average latency during the actual testing process was approximately 3 ms. With time, the BMI160 may experience drift, causing body parts to face the wrong direction after some time. Therefore, a calibration of the wearable device was required every 10 min.

The participants sequentially completed seven different types of work tasks under voice prompts. Each work task corresponds to a category of basic working postures. A rest period was scheduled between the fourth and fifth tasks for device reset. Table 3 displays the duration of each operation task. The experiment involved a participant with a mechanical engineering background familiar with assembly processes. The participant’s height is 1580 mm, and weight is 55 kg.

### 3.3. Operation Posture Recognition Method

**Figure 6:**
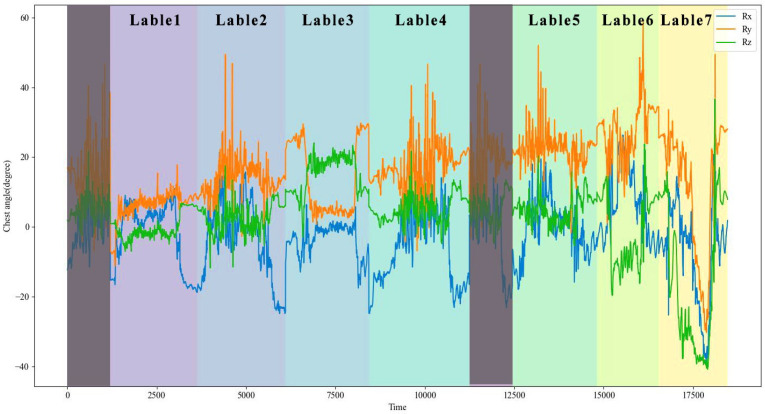
Chest joint angle over time, each color represents a category of working posture, the gray area is the excluded periods.

After signal processing, the experiment data were organized as the cumulative displacement of six joints and the joint angles of six joints over time. In preparation for posture recognition, removing the preparation and rest periods and labeling the remaining periods with corresponding posture labels is necessary. As shown in Figure 6, Taking the curve of chest joint angle over time as an example, the gray area in the graph represents the excluded periods. In the remaining periods, each color represents a category of working posture. 

**Figure 7:**
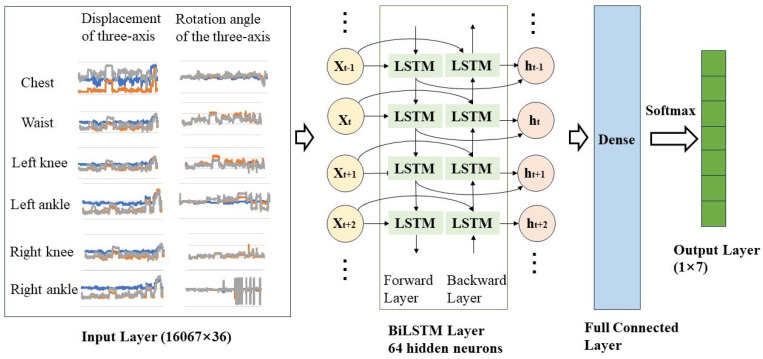
The operation posture recognition network structure.

BiLSTM is a deep learning model suitable for sequential data, and particularly effective for data with a temporal structure, such as time series. BiLSTM effectively captures contextual relationships and long-term dependencies in sequential data by combining forward and backward information. In recent years, the BiLSTM model has been commonly applied in research on IMU-based human posture recognition, demonstrating excellent recognition performance. Based on the experiment data, the BiLSTM model was used to recognize the seven basic operation postures: standing posture, slightly bending posture, deep bending posture, half-squatting posture, squatting posture, sitting posture, and supine posture. The operation posture recognition network structure is shown in Figure 7. Labeled experimental data were transformed into the dataset using a sliding window technique. The window length is 50 and the sliding size is 5; a total of 16,067 labeled samples were obtained for training. The input features included the displacement and rotation angles of six joints (chest, waist, left hip, right hip, left ankle, right ankle) in the XYZ direction, resulting in 36 features. The input layer of the network module is a 16,067 × 36 matrix. The input sequence is processed by two separate LSTM layers, each observing the sequence in both the forward and backward directions. The number of hidden neurons in each LSTM layer is 64. The input time-series data first pass through the forward layer. For each time step, the forward LSTM unit updates its internal state and produces an output. Similarly, the input sequence data also go through the backward layer. For each time step, the backward LSTM unit updates its internal state and produces an output. The outputs from both the forward and backward directions are merged. The merged representation is then passed to a fully connected layer. Finally, it is fed into an output layer for classification, using the softmax activation function to generate a probability distribution over the classes. The outputs of multiple neurons are mapped to the range of 0–1 to obtain the predicted probability distribution, which represents the probability of belonging to each category and enables posture prediction. This model was compiled using the cross-entropy loss function and adaptive moment estimation (Adam) optimizer. We divided the dataset into training and testing sets in a 4:1 ratio, the random state was set to 42. The epochs and batch size were set to 10 and 32. The initial learning rate was set as 0.001. L2 regularization with a dropout of 0.5 was selected to prevent overfitting of the model.

### 3.4. Operation Posture Recognition Result

**Figure 8:**
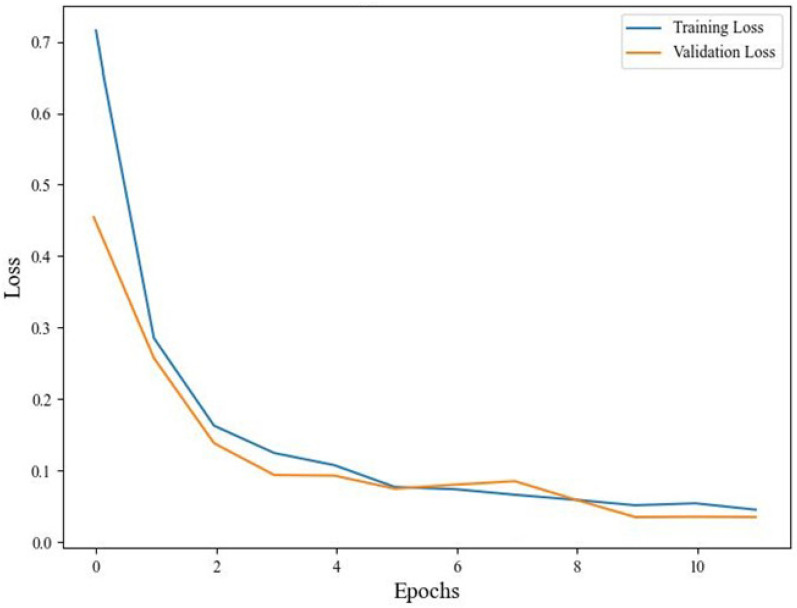
Training and validation loss.

The offline test was conducted on a workstation with an Intel Core i7-1165G7 CPU and NVIDIA GeForce MX 450 GPU. To reduce the random effects of the training tests, the sample order was randomly shuffled and the training test was repeated five times. The average training time was 75.08 s. Figure 8 shows the training and validation loss as the number of iterations increases. It can be observed that the loss curves of the training set and validation set tend to flatten after the 8th iteration. After the 10th generation, the test set loss remained stable below 0.05.

After the test, the average accuracy of posture prediction was 98.24%. The posture prediction transition time, including data preprocessing time and inference time, is 31 ms. Table 4 shows the accuracy, recall, and F-score of each posture prediction result. Each calculation result in the table is the average of 5 tests.

**Figure 9:**
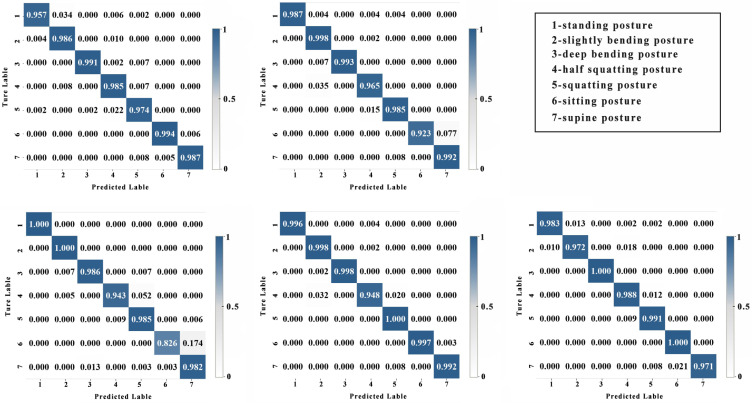
The confusion matrix from five tests.

The results are summarized in Table 4, which shows that (1) the precision of the seven postures are all above 96%, with the highest precision for the bending posture at 99.74% and the lowest for the squatting posture at 96.80%; (2) the recall of the seven postures are all above 96%, with the highest recall for the bending posture at 99.45% and the lowest for the half-squatting posture at 96.57%; (3) the F-score of the seven postures are all above 97%, with the highest F-score for the bending posture at 99.56% and the lowest for the half-squatting posture at 97.42%. Overall, the recognition performance is best for the deep bending posture, while the recognition performance for the half-squatting and squatting postures is relatively poor. Figure 9 shows the distribution of the test set confusion matrix from five tests. From the confusion matrix, it can be visually observed that the model performs well in classifying most postures. In comparison, the standing posture and half-squatting posture are more prone to be misclassified as a slightly bending posture.

The comparison of the results with existing research is shown in Table 5. In aspects of accuracy and time cost, we compared our work with other IMU-based human posture recognition works. The number of recognition classes and the number of IMUs are also shown in the table. In terms of accuracy, our work achieved a 98% accuracy for the classification of seven postures using six trackers, placing it in a relatively high position compared to similar studies. Regarding the time cost, we took into account the posture prediction transition time (including data preprocessing time and inference time), as well as the IMU sampling rate. While our method does not match the performance of the approach described in reference [28], we were able to identify a greater number of postures with more considerable accuracy.

## 4. Discussion

This paper focuses on operation posture collection and recognition based on low-cost IMU. The proposed method is available, and the accuracy of basic posture classification recognition is satisfactory. Integrating more features and employing more complex machine learning models may result in higher recognition accuracy, but it also comes with relatively higher time costs. Based on the data in this experiment, we compared the recognition accuracy of the LSTM and BiLSTM models. We conducted five training and testing times and calculated the average accuracy of classification on the test set. The average accuracy of LSTM is 95.81%, while the average accuracy of BiLSTM is 98.24%. Compared to LSTM, BiLSTM has a greater advantage in basic posture classification for assembly tasks. However, due to the complexity of the model, BiLSTM requires longer training time. In our test, LSTM took 34.82 s and BiLSTM took 75.08 s. However, the difference in prediction time between the two models is not significant: LSTM took 15ms and BiLSTM took 31 ms.

**Figure 10:**
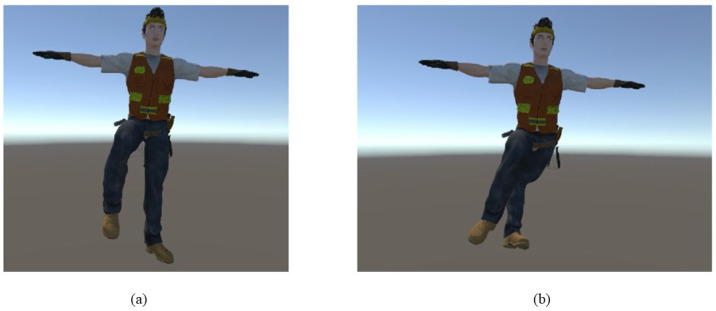
The sudden posture distortion when the network signal is unstable. (**a**) The normal posture. (**b**) The abnormal posture.

Using wireless network transmission of data can enhance convenience, but it may lead to sudden posture distortion when the network signal is unstable, as shown in Figure 10. The occurrence of abnormal postures is related to the network signal quality. In the experimental environment, the occurrence of anomalies is rare (1–2 times/10 min) and quickly recovers to normal. However, in environments with poorer signal quality, it can be foreseen that sudden abnormal postures will affect the observation of operation movements and the accuracy of posture recognition to some extent. How to identify and ignore exceptional signals is a research question that needs to be further studied. Because abnormal postures often manifest as sudden drifts in joint positions, a possible solution is to set a threshold for joint position changes and identify abnormal postures accordingly. Alternatively, based on gathering a sufficient number of data samples, machine learning models can be employed to differentiate between normal and abnormal states. We will attempt to address this issue in future work.

## 5. Conclusions

For the demand for operation posture recognition in the Industry 4.0 era, this paper explores a low-cost method for collecting assembly actions and recognizing assembly postures based on IMU. The study includes the following aspects:

A low-cost human motion collection system based on IMU has been proposed. The BMI160 inertial measurement module is combined with the ESP8266 communication module to create the motion collection tracker. Motion signals are transmitted via WiFi to the computer to obtain sensor pose information. The tracker is assigned to the corresponding joint positions of the digital human body based on the actual wearing position. Real-time calculation of human posture is performed by combining the hierarchical relationship of the human body skeleton. The Unity development platform receives human motion information and presents synchronized online visualization through a 3D digital human model;

We experimentally validate the feasibility of the action collection scheme. We have simulated various assembly tasks in a virtual reality environment and collected motion information for six joints of the subjects: chest, waist, left knee, left ankle, right knee, and right ankle, which included the rotation angles around XYZ directions and the displacement in XYZ directions. The BiLSTM model was used to identify seven common assembly postures: standing posture, slightly bending posture, deep bending posture, half-squatting posture, squatting posture, sitting posture, and supine posture. The model performs well in classifying these operation postures.

Based on the experiment results, the system could serve as a low-cost solution for the basic operation posture recognition of operation tasks. Subsequent research will focus on enhancing the operation posture recognition system and testing it in real factory environments.

## Figures and Tables

**Table 1:** Comparison of prices and performance of common IMUs.

Ref.	IMU Models	Price (1 IMU)	Sampling Rate	Acc. Noise [µg/ $\sqrt{\mathrm{Hz}}$ ]	Gyro. Noise [°/s/ $\sqrt{\mathrm{Hz}}$ ]	Interface	Battery Life
[10]	Xsens MTw Awinda	$437.2	120 Hz	200	0.01	Wireless 2.4 GHz	6 h
[10]	MetaMotionR	$98.4	100 Hz	300	0.007	Bluetooth LTE 2.4 GHz	1 to 14 days
[11,12]	NG IMU	$273.3	50 Hz/100 Hz	NA	NA	Preconfigured Wi-Fi router	4 to 12 h
[13]	MetaMotionC	$75	NA	300	0.007	Bluetooth 4.0	1 to 14 days
[14]	Shimmer3	$945.5	51.2 Hz	72.5	0.007	Bluetooth	NA
[15]	InvenSense MPU-9250	$10.9	120 Hz	300	0.01	I^2^ C−SPI	NA
	This work	$3.60	100 Hz	300	0.007	Wireless 2.4 GHz	6 h

**Table 2:** The basic postures.

Lable	Gesture Name	Gesture Picture	Torso Bending Angle (°)	Hip Joint Bending Angle (°)	Knee Joint Bending Angle (°)
1	standing posture	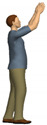	−15–15	−17–5	−3–5
2	slightly bending posture	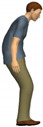	15–40	5–50	5–60
3	deep bending posture	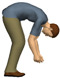	40–84	70–116	5–70
4	half squatting posture	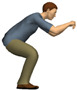	15–40	50–116	60–100
5	squatting posture	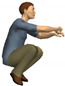	15–40	50–116	100–150
6	sitting posture	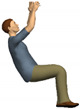	−15–15	30–70	−3–5
7	supine posture	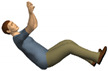	−5–40	50–100	5–60

**Table 3:** The duration of each operation task.

Task Name	Duration (ms)
Preparation	60,000
Installing bolts in standing posture	121,800
Installing bolts in slightly bending posture	123,250
Installing bolts in deep bending posture	116,550
Installing bolts in half squatting posture	140,600
Break time	60,000
Installing bolts in squatting posture	118,350
Installing bolts in sitting posture	86,500
Installing bolts in supine posture	96,300

**Table 4:** Classification accuracy of the test set.

Posture Category	Precision	Recall	F-Score
1—standing	0.9940	0.9859	0.9905
2—slightly bending	0.9764	0.9909	0.9800
3—folding	0.9974	0.9945	0.9956
4—half-squatting	0.9832	0.9657	0.9742
5—squatting	0.9680	0.9871	0.9773
6—sitting posture	0.9936	0.9721	0.9823
7—supine posture	0.9752	0.9850	0.9859

**Table 5:** Comparison of the human posture recognition results.

Ref.	Model	Accuracy	Prediction Time	Sampling Rate	Recognition Classes	Number of IMUs
[29]	ANN	82.74%	NA	50 Hz	8	2
[13]	CLN model	90.00%	NA	NA	7	5
[12]	LSTM	94.44%	170 ms	100 Hz	3	4
[28]	DCNN-BiLSTM	96.00%	8.47 ms	120 Hz	4	3
[30]	RBF-SVM	97.35%	120 ms	62 Hz	8	3
[11]	LSTM-RNN	99.00%	277 ms–488 ms	50 Hz	7	4
This work	BiLSTM	98.24%	31 ms	100 Hz	7	6

## Data Availability

The original experiment data are available.
